# Culturable diversity and antimicrobial activity of Actinobacteria from marine sediments in Valparaíso bay, Chile

**DOI:** 10.3389/fmicb.2015.00737

**Published:** 2015-07-28

**Authors:** Fernanda P. Claverías, Agustina Undabarrena, Myriam González, Michael Seeger, Beatriz Cámara

**Affiliations:** Laboratorio de Microbiología Molecular y Biotecnología Ambiental, Departamento de Química and Centro de Biotecnología Daniel Alkalay Lowitt, Universidad Técnica Federico Santa MaríaValparaíso, Chile

**Keywords:** marine Actinobacteria, phylogenetic diversity, antimicrobial activity, Valparaiso bay, *Nocardiopsaceae*

## Abstract

Marine-derived Actinobacteria are a source of a broad variety of secondary metabolites with diverse biological activities, such as antibiotics and antitumorals; many of which have been developed for clinical use. Rare Actinobacteria represent an untapped source of new bioactive compounds that have been scarcely recognized. In this study, rare Actinobacteria from marine sediments were isolated from the Valparaíso bay, Chile, and their potential to produce antibacterial compounds was evaluated. Different culture conditions and selective media that select the growth of Actinobacteria were used leading to the isolation of 68 bacterial strains. Comparative analysis of the 16S rRNA gene sequences led to identifying isolates that belong to the phylum *Actinobacteria* with genetic affiliations to 17 genera: *Aeromicrobium, Agrococcus, Arthrobacter, Brachybacterium, Corynebacterium, Dietzia, Flaviflexus, Gordonia, Isoptericola, Janibacter, Microbacterium, Mycobacterium, Ornithinimicrobium, Pseudonocardia, Rhodococcus, Streptomyces*, and *Tessaracoccus*. Also, one isolate could not be consistently classified and formed a novel phylogenetic branch related to the *Nocardiopsaceae* family. The antimicrobial activity of these isolates was evaluated, demonstrating the capability of specific novel isolates to inhibit the growth of Gram-positive and Gram-negative bacteria. In conclusion, this study shows a rich biodiversity of culturable Actinobacteria, associated to marine sediments from Valparaíso bay, highlighting novel rare Actinobacteria, and their potential for the production of biologically active compounds.

## Introduction

Bioactive compounds are increasingly required for diverse biotechnological applications. One of the main targets is focused on the discovery of new drugs, such as antibiotics, to combat antibiotic resistant pathogens (Payne et al., 2007). The problem of multidrug-resistant bacteria on a global scale is an important challenge. Multi-drug resistant bacteria are generally nosocomial but one of the most relevant cases, methicillin-resistant *Staphylococcus aureus* (MRSA), is also present in community settings, the latter being increasingly more prevalent and virulent (Enright, 2003; Chambers and DeLeo, 2009). As a result, there is a continuous demand to discover new antibiotic compounds. Despite all chemically synthetic efforts, natural environments are still the best supplier for these novel compounds (Fenical and Jensen, 2006; Bull and Stach, 2007). It is crucial that new groups of microbes from unexplored habitats are pursued as sources of novel antibiotics and other bioactive compounds (Magarvey et al., 2004; Goodfellow and Fiedler, 2010). Currently, the phylum *Actinobacteria*, especially actinomycetes (order *Actinomycetales*), represent the most prominent group of microorganisms for the production of bioactive compounds, notably antibiotics and antitumor agents (Stach et al., 2003; Goodfellow and Fiedler, 2010). Close to 40% of all microbial bioactive secondary metabolites derive from Actinobacteria, where approximately 80% of them are produced by the genus *Streptomyces* (Goodfellow and Fiedler, 2010; Bérdy, 2012). In fact, two of the four new classes of antibiotics discovered in recent years have been derived from actinobacterial strains (Raja et al., 2003; Hardesty and Juang, 2011).

Marine ecosystems are unique environments, characterized by high salinity and pressure, low temperatures and variable oxygen concentrations (Bull et al., 2000). All these conditions have generated an evolutionary pressure on marine microorganisms, differentiating them from their terrestrial counterparts, which is likely to be reflected on the genetic and metabolic diversity of marine microorganisms (Lam, 2006; Manivasagan et al., 2013). Although the ecological functions of Actinobacteria in marine sediments is largely unknown (Duran et al., 2015), they may play an important ecological role in the biogeochemical cycle due to their capacity to break down polymeric substances and turnover organic matter (Stevens et al., 2007). Furthermore, it has been described that Actinobacteria act as symbionts in marine sponges (Hentschel et al., 2012). Marine Actinobacteria have been described as an emerging source for novel bioactive molecules (Fenical and Jensen, 2006; Lam, 2006; Bull and Stach, 2007; Waters et al., 2010; Imhoff et al., 2011; Zotchev, 2012). The importance of cultivating these microorganisms is indispensable for a viable opportunity to biodiscovery (Joint et al., 2010). From 2005 to date, several novel genera of the so-called rare Actinobacteria have been discovered from marine environments, of which 13 of them have been isolated from marine sediments. These include *Salinispora* (Maldonado et al., 2005a), *Demequina* (Yi et al., 2007), *Aestuariimicrobium* (Jung et al., 2007), *Sciscionella* (Tian et al., 2009b), *Marinactinospora* (Tian et al., 2009a), *Paraoerskovia* (Khan et al., 2009), *Marisediminicola* (Li et al., 2010), *Miniimonas* (Ue et al., 2011), *Spinactinospora* (Chang et al., 2011), *Sediminihabitans* (Hamada et al., 2012), *Flaviflexus* (Du et al., 2013), *Mariniluteicoccus* (Zhang et al., 2014a), and *Halopolyspora* (Lai et al., 2014). Rare Actinobacteria are considered to be those strains that are less likely to be cultivated by conventional methods (Lazzarini et al., 2001; Baltz, 2006).

Investigations focused on marine actinobacterial isolates from Chile have been rather scarce (Jiang et al., 2010; Park et al., 2013), considering the extensive coast with a wide range of latitudes that Chile offers. Underexplored marine ecosystems, such as the Valparaiso Bay, provide access to novel microbial diversity, which is a crucial characteristic when pursuing novel biologically active molecules. In fact, a novel compound denominated thienodolin with a unique mechanism of action has been isolated from a *Streptomyces* strain derived from marine sediments in Valparaíso (Park et al., 2013). However, the precise isolation conditions were not described. As far as we know, the isolation of marine Actinobacteria in central Chile has not been previously described. Consequently, the isolation and characterization of novel Actinobacteria from marine sediments of the Valparaíso bay in Central Chile provides the basis for assessing the culturable biodiversity as well as the potential of these isolates to possess antibacterial activity.

## Materials and methods

### Sediment sampling

**Figure 1:**
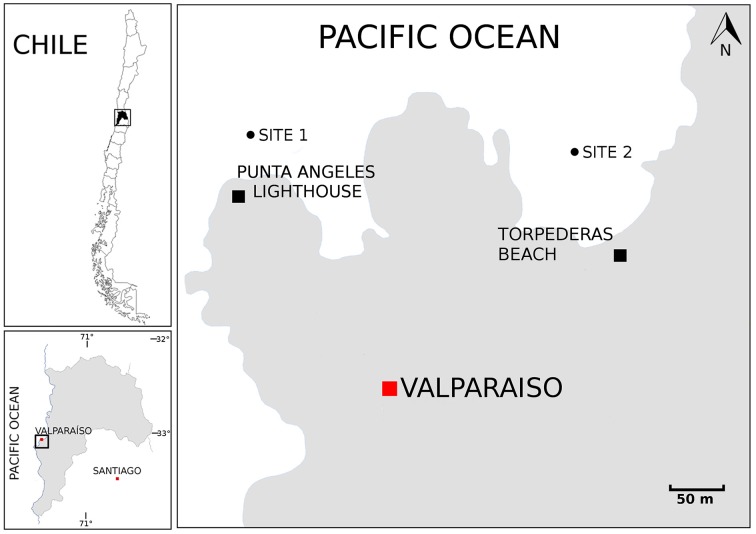
**Map showing the sampling locations**. Map of Chile. Boxed region is enlarged in the left bottom panel to emphasize the Valparaíso Region and boxed region is enlarged in the right panel to emphasize the sampling sites located near Punta Ángeles Lighthouse and Torpederas Beach.

A total of six sediment samples were collected from the bay of Valparaíso, Central Chile, during late summer in March 2013 (Figure 1). During this time of the year, the temperature of the surface seawater is in the range from 14 to 17°C. The temperature at 10 and 30 m depth ranged between 12.5 and 11°C, according to CTD measurements at the monitoring station located in Valparaíso bay. Subtidal zones from Torpederas Beach (33°1′ 11.05″ S 71°38′ 43.25″ W) and Punta Ángeles Lighthouse site (33°1′ 12.21″ S 71°38′ 56.41″ W) were sampled at three different depths: approximately (from 6.7 to 29.4 m) with salinity ranging from 31.85 to 32.77 g/mL. Marine surface sediments (0–5 cm) were transferred to sterile conical tubes (50 mL) with the help of scuba divers. Samples were transported to the laboratory on ice (≤1 h) and stored overnight at 4°C until use. Samples were used for streaking out primary plates during the following 2 days.

### Isolation of actinobacterial strains

Five selective media: modified M1 (Mincer et al., 2002), modified ISP2 (Shirling and Gottlieb, 1966; Magarvey et al., 2004), modified R2A (Difco), Marine agar 2216 (Difco), and modified NaST21Cx (Magarvey et al., 2004) were prepared with artificial sea water (ASW) (Kester et al., 1967), with exception of Marine agar 2216. All isolation media were supplemented with 0.2 μm pore size filtered cycloheximide (100 μg/mL) to inhibit fungal growth and nalidixic acid (25 μg/mL) to inhibit the growth of Gram-negative bacteria to favor the growth of slow-growing Actinobacteria. The prepared media were used for the isolation of actinobacterial strains by either plating directly or using 100 μL of serial dilutions 10^−4^ and 10^−6^ from the sediment samples. Direct plating was carried out by directly streaking sediment samples onto the agar using a sterile loop. Inoculated plates were incubated at 20°C or 30°C for 6–12 weeks and colonies were selected based on morphology. After successive transfers, pure isolates grown in modified TSA media (Difco) with ASW at 30°C were frozen at −80°C in 10% TSB medium (Bacto) with ASW using 20% glycerol for long-term storage.

### Molecular PCR screening of isolates

To identify potential Actinobacteria within the isolated strains, an initial PCR screening was accomplished using S-C-Act-0235-a-S-20 and S-C-Act-0878-A-19 primers specific for amplification of V3 to V5 regions of 16S rRNA gene from Actinobacteria (Stach et al., 2003). Genomic DNA was prepared as described previously (Moore et al., 2004). Each PCR reaction contained 1 μL of genomic DNA, 12.5 μL of GoTaq Green Master Mix and 0.6 μM of each primer in a final reaction volume of 25 μL. The reaction was started with an initial denaturation at 95°C for 5 min followed by 35 cycles of denaturation at 95°C for 1 min, annealing at 70°C for 1 min and extension at 72°C for 1.5 min, with a final extension at 72°C for 10 min. Amplicons were fractioned in 2% agarose gel electrophoresis and subsequently revealed with SYBR Green (E-gel, Invitrogen).

### Molecular identification and phylogenetic analysis

For 16S rRNA gene amplification, universal primers 27F and 1492R (Lane, 1991) were used in the PCR reaction. The reaction mix (50 μL) contained 1 μL of genomic DNA, 25 μL of GoTaq Green Master Mix and 0.2 μM of each primer. The reaction was started with an initial denaturation at 95°C for 5 min followed by 30 cycles of denaturation at 95°C for 1 min, annealing at 55°C for 1 min and extension at 72°C for 1.5 min, with a final extension at 72°C for 10 min. Products were quantified and submitted for purification and sequencing to Macrogen Inc. (Seoul, Korea). For partial sequencing the universal primer 800R was used, whereas the universal primers 27F, 518F, 800R, and 1492R were used for almost complete sequencing. The genus-level affiliation of the sequences was validated using sequences from the BLAST server from National Center for Biotechnology Information (NCBI). Sequence alignment and phylogenetic analysis were performed using Vector NTI v10 software package (Invitrogen). Tree construction based on the V1 to V9 region of the 16S rRNA gene sequences was conducted using the neighbor joining algorithm (Saitou and Nei, 1987) with bootstrap values based on 1000 replications (Felsenstein, 1987) using the MEGA software version 6.0 (Tamura et al., 2013). The 16S rRNA gene sequences of the novel isolates were deposited in GenBank under the accession numbers KM406755-KM406774. In addition, partial 16S rRNA gene sequences of the remaining isolates were deposited in GenBank under the accession numbers KT152237-KT152284.

### Antibacterial activity screening

Representative actinobacterial isolates from Valparaíso were screened for antimicrobial activity using a qualitative cross streak method (slightly modified from Haber and Ilan, 2013). For streptomycete-like strains, four different media were used for growing each isolate, i.e., Marine agar 2216, modified ISP2, modified ISP3, and modified TSA. For non streptomycete-like strains, both modified ISP2, and modified TSA media were used. Actinobacterial cultures were inoculated as a single middle line dividing the agar plate into two equal-sized halves. Plates were incubated at 30°C for 5–10 days until a visibly well-grown bacterial dividing line was observed. Five laboratory test strains (*Staphylococcus aureus* NBRC 100910^T^, *Listeria monocytogenes* 07PF0776, *Salmonella enterica* subsp enterica LT2^T^, *Escherichia coli* FAP1, and *Pseudomonas aeruginosa* DSM50071^T^) were used to test their susceptibility toward the Actinobacteria. Test strains were grown overnight on LB medium at 37°C. A 10 μl aliquot of an overnight culture of test bacteria was inoculated on the plate, close to the Actinobacteria line. For a homogenous seeding, test bacteria were streaked outwards to the border of the plate and subsequently inwards, perpendicular to the Actinobacteria line, for a total of five streaks. A maximum of three test bacteria were inoculated on one single plate. Plates were incubated at 37°C and inhibition was registered both after 24 and 48 h. Inhibitions were seen as part of the test bacteria line, where the test bacterium did not grow. Inhibitions were visualized and ranked as: −, no inhibition; +∕−, attenuated growth of test strain; +, < 50% growth inhibition (less than half of the bacterial line was inhibited); ++, 50% growth inhibition (half of the bacterial line was inhibited); +++, >50% growth inhibition (more than half of the bacterial line was inhibited). Duplicates were performed using an internal control with one of the test strains.

## Results

### Isolation of actinobacteria from marine sediments of valparaíso

**Figure 2:**
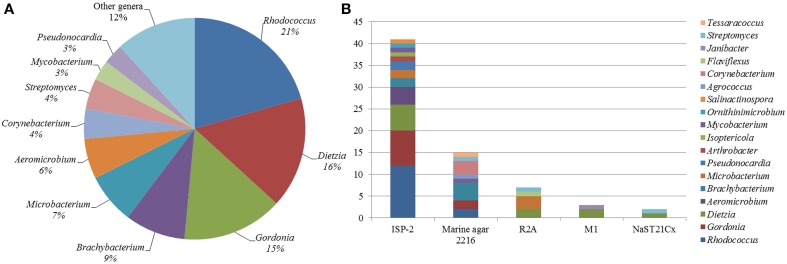
**Diversity of culturable Actinobacteria from Valparaíso. (A)** Pie chart representation of the percentage frequency of actinobacterial genera within the total number of isolates. **(B)** Number of actinobacterial isolates according to the culture media used.

**Figure 3:**
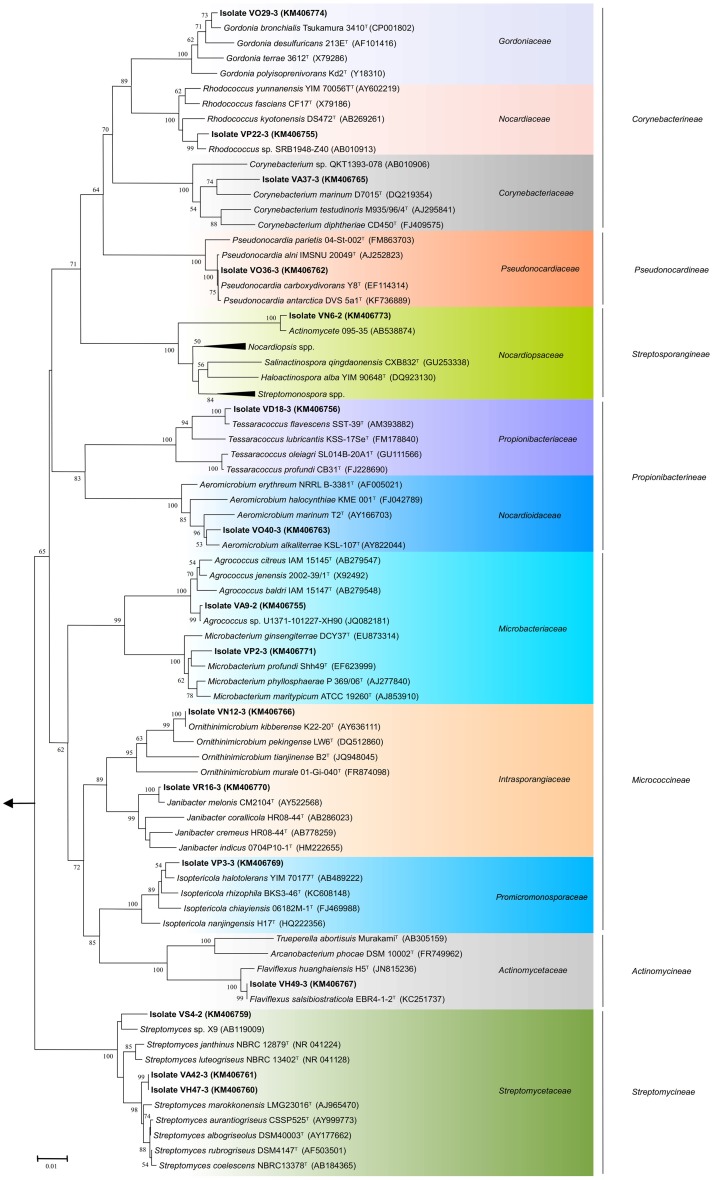
**Phylogenetic tree of actinobacterial isolates from Valparaíso bay that belong to the suborders *Actinomycineae*, *Corynebacterineae*, *Micrococcineae*, *Propionibacterineae*, *Pseudonocardineae*, *Streptomycineae*, *Streptosporangineae* and closely related representative species**. The tree was constructed based on the V1 to V9 region of the 16S rRNA gene sequences using the neighbor-joining method with the percentage of bootstrap replicates (1000 resamplings) supporting the proposed branching order shown at consistent nodes (values below 50% not shown). Gene sequence positions 101–1426 were considered, according to the *Escherichia coli* K12 16S rRNA gene sequence numbering. Arrow points to the outgroup *E. coli* K12 (AP012306). The GenBank accession numbers of 16S rRNA sequences are given in parentheses. Scale bar corresponds to 0.01 substitutions per nucleotide positions.

A total of 68 actinobacterial strains were isolated from six marine sediment samples obtained from two sites located near the Punta Ángeles Lighthouse (site 1) and Torpederas Beach (site 2) in Valparaíso Bay in March 2013 (Figure 1). NCBI nucleotide BLAST of the partial 16SrRNA genes sequences (approximately 600 bp) revealed that the isolated strains belong to the phylum *Actinobacteria* with genetic affiliations to 18 genera, representing seven suborders and 16 families: *Actinomycetaceae, Corynebacteriaceae, Dermabacteraceae, Dietziaceae, Gordoniaceae, Intrasporangiaceae, Microbacteriaceae, Micrococcaceae, Mycobacteriaceae, Nocardiaceae, Nocardioidaceae, Nocardiopsaceae, Promicromonosporaceae, Propionibacteriaceae, Pseudonocardiaceae*, and *Streptomycetaceae*. The most abundant isolates were affiliated to the genera *Rhodococcus, Dietzia*, and *Gordonia* (Figure 2A), all belonging to the suborder *Corynebacterineae* (Figure 3). Only six isolates formed powdery colonies with well-developed substrate and aerial mycelia and were considered to be streptomycete-like strains. These strains were associated with the genera *Salinactinospora, Streptomyces*, and *Pseudonocardia*. Although the strains have these common characteristics, single colonies have distinctive features including different times of growth and sporulation. For the strain belonging to the genus *Isoptericola*, a well-developed yellow substrate mycelia was observed but no aerial mycelia or spore, similar to *Isoptericola salitolerans* TRM F109^T^ (Guan et al., 2013). The remaining isolates formed white, yellow, orange, and red pigmented colonies with no hyphae.

Five culture media were used in order to isolate a diversity of actinobacterial strains. The use of different culture media had an important effect on the total number of Actinobacteria recovered from the sediment samples. The greatest number of isolates as well as diversity of Actinobacteria was isolated with modified ISP-2 media yielding 41 strains affiliated to 12 genera, of which six of them were exclusively isolated in this media. The media that followed in number of actinobacterial isolates and diversity are Marine agar 2216, modified R2A, modified M1 and modified NaST21Cx (Figure 2B). There were substantially more actinobacterial strains isolated from site 1 (52 isolates) in comparison to the 16 isolates from site 2 (Figure 1). In fact, sample V2 from site 1, collected at a depth of 19.2 m, contributed to the highest number of isolates (Figure S1), yielding 42 actinobacterial strains affiliated to 10 genera.

#### Phylogenetic diversity of isolated actinobacteria

The almost complete sequencing of the 16S rRNA gene was performed to 20 representative actinobacterial strains isolated from these sediment samples. Comparison of the V1 to V9 region of the 16S rRNA gene sequences (between 1221 and 1321 nucleotides) of 16 of the 20 representative strains was used to construct phylogenetic trees. Twelve of the twenty representative isolates shared 99.1–99.9% sequence similarities with the closest type strains. Although there is no established sequence similarity cut-off to assign new bacterial species, six strains exhibited low sequence similarities with validly described species based on a genus-specific literature search; suggesting that these strains could represent novel taxons within the phylum *Actinobacteria*. These putative novel isolates were affiliated to the genera: *Agrococcus* (suborder *Micrococcineae*), *Corynebacterium* (suborder *Corynebacterineae*), *Microbacterium* (suborder *Micrococcineae*), *Rhodococcus* (suborder *Corynebacterineae*), *Salinactinospora* (suborder *Streptosporangineae*), and *Streptomyces* (suborder *Streptomycineae*), representing four different suborders (Figure 3). Isolate VS4-2 presented 98.3% sequence similarity to *Streptomyces janthinus* NBRC 12879^T^, possibly indicating a new taxon within the *Streptomycetaceae* family. The isolates VA42-3 and VH47-3 form a separate cluster from close relatives of the genus *Streptomyces* (Figure 3). Interestingly, isolate VN6-2 exhibited 93.9% sequence similarity to *Salinactinospora qingdaonensis* CXB832^T^, which was isolated from a salt pond in Qingdao, China (Chang et al., 2012). Phylogenetic analysis revealed that isolate VN6-2 forms a distinct branch together with an unpublished strain from marine sediment, actinomycete 095-35, which is well supported (Figure 3). Together with the low sequence similarity, VN6-2 appears to represent a distinct taxon related with the *Nocardiopsaceae* family. The phylogenetic analysis presented implies a considerable diversity of culturable Actinobacteria within sea sediments from Valparaíso bay, including the isolation of rare Actinobacteria.

### Antibacterial activity of actinobaterial isolates

All isolates from the *Streptomyces* genus and one representative strain of each other genus were evaluated for antibacterial activity. Isolates were tested for antibacterial activities against the Gram-negative bacteria *Escherichia coli* (ECO), *Pseudomonas aeruginosa* (PSAU) and *Salmonella enterica* (SAEN), and the Gram-positive bacteria *Staphylococcus aureus* (STAU) and *Listeria monocytogenes* (LIMO). Isolates with antibacterial activity belonged to 10 of the selected strains representing 18 genera (Tables 1, 2).

**Table 1:** **Antimicrobial activity of streptomycete-like strains**.

**Strain**	**Closest type strain (Accession N°) (% Identity)**	**Media**	**Laboratory test strains^a^**				
			**SAEN**	**STAU**	**PSAU**	**ECO**	**LIMO**
VH47-3	*Streptomyces albogriseolus* DSM 40003^T^ (AY177662) (99.41)	MA	−	−	−	−	−
		ISP2	+∕−	−	−	−	+
		ISP3	−	−	−	−	−
		TSA	−	−	−	−	−
VA42-3	*Streptomyces aurantiogriseus* CSSP 525^T^ (AY999773) (99.36)	MA	−	−	−	−	−
		ISP2	+∕−	−	−	−	−
		ISP3	−	−	−	−	−
		TSA	−	−	−	−	−
VS4-2	*Streptomyces janthinus* NBRC 12879^T^ (AB184851) (98.32)	MA	−	+∕−	−	−	+
		ISP2	+∕−	+∕−	−	+∕−	+∕−
		ISP3	−	−	−	−	−
		TSA	−	−	−	−	−
VO36-3	*Pseudonocardia carboxydivorans* Y8^T^ (EF114314) (99.86)	MA	−	−	−	−	−
		ISP2	+∕−	+∕−	−	−	−
		ISP3	−	−	−	−	−
		TSA	−	+∕−	−	−	+++
VN6-2	*Salinoactinospora qingdaoensis* CXB 832^T^ (GU253338) (93.92)	MA	−	−	−	+++	+++
		ISP2	−	+∕−	−	−	+∕−
		ISP3	−	−	−	−	−
		TSA	−	++	+∕−	+++	+++

a *SAEN, Salmonella enterica; STAU, Staphylococcus aureus; PSAU, Pseudomonas aeruginosa; ECO, Escherichia coli; LIMO, Listeria monocytogenes; −, no inhibition; +∕−, attenuated growth of test strain; +, < 50% growth inhibition; ++, 50% growth inhibition; +++, >50% growth inhibition. Media ISP2, ISP3, and TSA were prepared with ASW*.

**Table 2:** **Antimicrobial activity of non streptomycete-like strains**.

**Strain**	**Closest type strain (Accession N°) (% Identity)**	**Media**	**Laboratory test strains^a^**				
			**SAEN**	**STAU**	**PSAU**	**ECO**	**LIMO**
VI37-3	*Dietzia maris* AUCM A-593^T^	ISP2	nd	+∕−	nd	−	−
	(X79290) (99.92)	TSA	−	−	−	−	+∕−
VP3-3	*Isoptericola halotolerans* NRBC 104116^T^	ISP2	nd	++	nd	+	++
	(AB489222) (99.15)	TSA	−	+∕−	−	+	+∕−
VA16-3	*Mycobacterium vaccae* ATCC15483^T^	ISP2	−	−	−	−	−
	(X55601) (99.50)	TSA	−	−	−	−	−
VR16-3	*Janibacter melonis* CM2104^T^	ISP2	NG				
	(AY522568) (99.58)	TSA	−	+∕−	−	−	−
VP2-3	*Microbacterium profundi* Shh49^T^	ISP2	nd	+∕−	nd	−	+++
	(EF623999) (98.56)	TSA	−	+∕−	−	−	+∕−
VO29-3	*Gordonia bronchialis* DSM43247^T^	ISP2	−	+∕−	+∕−	−	−
	(CP001802) (99.01)	TSA	−	+∕−	−	−	++
VN12-3	*Ornithinimicrobium kibberense* K22-20^T^	ISP2	nd	+∕−	nd	−	+∕−
	(AY636111) (99.78)	TSA	−	−	−	−	−
VR7-2	*Brachybacterium conglomeratum* J 1015^T^	ISP2	NG				
	(AB537169) (99.63)	TSA	−	+∕−	−	−	−
VO30-3	*Arthrobacter phenanthrenivorans* Sphe3^T^	ISP2	−	−	−	−	−
	(CP002379) (99.92)	TSA	+∕−	+	−	+++	+++
VP22-3	*Rhodococcus yunnanensis* YIM 70056^T^	ISP2	−	−	−	−	−
	(AY602219) (98.31)	TSA	−	−	−	+	−
VA37-3	*Corynebacterium marinum* D7015^T^	ISP2	NG				
	(DQ219354) (97.63)	TSA	−	−	−	−	−
V040-3	*Aeromicrobium alkaliterrae* KSL-107^T^	ISP2	−	++	−	−	++
	(AY822044) (99.08)	TSA	+∕−	++	+∕−	+∕−	++
VH49-3	*Flaviflexus salsibiostraticola* EBR4-1-2^T^	ISP2			NG		
	(KC251737) (99.85)	TSA	−	−	−	−	−
VA9-2	*Agrococcus baldri* IAM 15147^T^	ISP2	−	−	−	−	−
	(AB279548) (98.90)	TSA	−	−	−	−	+∕−
VD18-3	*Tessaracoccus flavescens* SST-39^T^	ISP2			NG		
	(AM393882) (99.64)	TSA	−	−	−	−	+∕−

a *SAEN, Salmonella enterica; STAU, Staphylococcus aureus; PSAU, Pseudomonas aeruginosa; ECO, Escherichia coli; LIMO, Listeria monocytogenes; −, no inhibition; +∕−, attenuated growth of test strain; +, < 50% growth inhibition; ++, 50% growth inhibition; +++, >50% growth inhibition. nd, not determined; NG, no growth. Media ISP2 and TSA were prepared with ASW*.

All streptomycete-like strains showed antibacterial activity against at least two of the five test strains (Table 1). Although most strains showed an attenuated growth effect on test bacteria, the strongest antibacterial effect was shown with the rare actinobacteria strain VN6-2 against both Gram negative (ECO) and Gram positive (LIMO) strains. Strain VO36-3 showed the strongest inhibitory effects against *L. monocytogenes*. The various culture conditions used showed a strong influence on the production of the antibacterial compounds by the isolates.

Fifteen non streptomycete-like strains were evaluated for antibacterial screening against *E. coli, P. aeruginosa, S. aureus, S. enterica*, and *L. monocytogenes*. Six strains showed at least one inhibitory effect (Table 2). Isolates VR16-3, VR7-2, VA37-3, VH49-3, and VD18-3 did not grow enough on ISP-2 media to perform the antibacterial assay. Hence it was not possible to evaluate the influence of other culture conditions. Both *S. aureus* and *L. monocytogenes* growth was inhibited when exposed to nine different actinobacterial genera. *P. aeruginosa* showed attenuated growth when exposed to two different actinobacterial genera. Isolates VP3-3, VP2-3, VO29-3, VO30-3, and VO40-3 showed the strongest inhibitory effects against *L. monocytogenes*.

## Discussion

Marine sediments may harbor a great diversity of culturable Actinobacteria (Bredholdt et al., 2007; Gontang et al., 2007; Solano et al., 2009; Yuan et al., 2014). In this study, a large culturable biodiversity of Actinobacteria was obtained from Valparaíso bay, Central Chile. This is unexpected since only a small number of samples (6) and primary agar plates (90) were used for isolation. Twenty representative isolates from Valparaíso bay that were studied in more detail comprise seven of the 14 suborders of the order *Actinomycetales*. The number of different genera isolated (18) from marine sediments of Valparaíso bay was within the range of other isolation studies from marine sediments (Bredholdt et al., 2007; Gontang et al., 2007; Solano et al., 2009; Yuan et al., 2014; Zhang et al., 2014b). Recently, Yuan et al. (2014) reported the diversity of actinobacterial isolates belonging to five suborders (14 genera) from sediments of the Arctic Ocean, using 10 sediment samples and 120 primary plates. The isolation of 25 actinobacterial genera belonging to eight suborders from 225 sediment samples of the Republic of Palau, were achieved using ≥675 primary plates (Gontang et al., 2007). Bredholdt et al. (2007) isolated Actinobacteria belonging to seven suborders starting from >2500 Actinobacteria with a small number of sediment samples (4). The different isolation procedures used in the studies mentioned above have to be acknowledged. The actinobacterial diversity in our study is relatively high, when considering the number of genera vs. the number of isolated actinobacterial strains. One possible explanation for such culturable diversity can be due to the hydrographic features (upwelling) present in Valparaíso bay. It is known that the upwelling phenomenon can contribute to transporting nutrients to the surface (Giovannoni and Stingl, 2005). In fact, upwelling regimes are related to the most biologically productive ecosystems in the ocean (Capone and Hutchins, 2013).

All genera uncovered in our study from Valparaiso bay have been previously isolated in diverse marine environments located in various regions of the world (Helmke and Weyland, 1984; Bruns et al., 2003; Chen et al., 2005; Gontang et al., 2007; Kageyama et al., 2007; Lee and Lee, 2008; Ben-Dov et al., 2009; Maldonado et al., 2009; Pimentel-Elardo et al., 2009; Abdelmohsen et al., 2010; Chang et al., 2011; Tian et al., 2013; Yu et al., 2013; Yuan et al., 2014), indicating that these genera are widely distributed in marine environments. *Streptomyces, Rhodococcus*, and *Micromonospora* seem to be readily cultured actinobacterial genera in marine sediments (Colquhoun et al., 1998; Maldonado et al., 2005b; Bredholdt et al., 2007; Duncan et al., 2015), however this observation relies on the influence of the culture methods used. In this study, isolates from the genus *Rhodococcus* were the most abundant (Figure 2A). One of these isolates (VP22-3) is probably a new *Rhodococcus* species. On the other hand, three representatives of the *Streptomyces* sp. were isolated, although this is not surprising since these microorganisms are susceptible of being cultivated under laboratory conditions (Fiedler et al., 2005; Maldonado et al., 2005b; Busti et al., 2006; Bredholdt et al., 2007; Duncan et al., 2015). One of the *Streptomyces* isolated in this study (strain VS4-2) is a good candidate to become a novel species, due to its relatively low sequence similarity to its closest type strain (Table 1). Although the other two streptomycete strains (VA42-3 and VH47-3) that interestingly form a distinct branch within the *Streptomyces* clade shared an identical 16S gene sequence (Figure 3), they are distinct strains due to their differences in morphological features. Under the same cultivating conditions, strain VA42-3 grows faster hence sporulates earlier than strain VH47-3. In this study, *Micromonospora* strains were not among the isolates, possibly due to the isolation procedures used. The taxonomic classification shown in Figure 3 is in agreement with previous reports (Zhi et al., 2009), with exception of the suborder *Actinomycineae*, which forms a distinct branch within the suborder *Micrococcineae*. However, this discordance has been seen in other studies using different methods (maximum parsimony and maximum likelihood) (Zhi et al., 2009). Along with the improvement of taxonomic techniques, the phylogenetic position of members of this suborder have been changed several times (Zhao et al., 2014).

The phylogenetic analysis for the suborder *Corynebacterineae* (Figure 3) provides evidence for the occurrence of novel taxa in the sea sediments of Valparaíso bay. Isolate VA37-3 appears to be a new species of *Corynebacterium*. Interestingly, the *Corynebacterium* isolate forms a distinct branch with a validly described marine species *C. marinum* D7015^T^ (Du et al., 2010), isolated from coastal sediments in Qingdao, China. Strains from this branch have been isolated from sediments across the Pacific Ocean and notably, do not group with the the validly described marine species *C. maris* Coryn-1^T^ isolated from the Red Sea (Ben-Dov et al., 2009). Something similar occurs with the *Rhodococcus* isolate VP22-3, which forms a cluster with a strain from deep sea sediments of Suruga Bay, Japan (Colquhoun et al., 1998). It is worthwhile noting that the highest similarities were shown with isolates derived from various marine environments: Antarctica, Artic, deep-sea, and coastal sediments, supporting a marine clade distinct from its closer relatives of terrestrial origin (data not shown). In the same way, our *Microbacterium* isolate VP2-3 groups together with *M. profundi* Shh49^T^ (Figure 3), isolated from the East Pacific. However, it does not form a cluster with the marine-derived *Microbacterium*, that is, *M. sediminis* YLB-01^T^ (Yu et al., 2013), *M. hydrothermale* 0704C9-2^T^ (Zhang et al., 2014c), and *M. marinum* H101^T^ (Zhang et al., 2012) that were isolated from the Indian Ocean. From an ecological point of view, some isolates derived from Valparaíso sea sediments seem to have a common ancestor with strains that are also isolated from the Pacific Ocean and have less relation to isolates derived from other oceans, such as the Indian Ocean. But to further explore the idea of an ocean-specific niche for microorganisms, a substantially higher number of strains from different geographical locations need to be isolated and phylogenetically characterized.

Many of the genera isolated in this study are considered to be rare Actinobacteria. The term “rare Actinobacteria” is usually used for strains that are less likely to be isolated than streptomycete strains (Jose and Jebakumar, 2013). In our study, these include *Agrococcus, Flaviflexus, Isoptericola, Janibacter, Ornithinimicrobium*, and *Tessaracoccus*. As an example, the genus *Flaviflexus* was recently proposed based on *F. huanghaiensis* H5^T^ that was isolated from sediment samples of the coastal area of Qingdao, China (Du et al., 2013). To date, this genus comprises only two species that also includes *F. salsibiostraticola* EBR4-1-2^T^ (Jin et al., 2014). Generally, these microorganisms have been isolated using pretreatments or complex enriching techniques (Jensen et al., 2005; Pathom-aree et al., 2006; Bredholdt et al., 2007; Solano et al., 2009). Nevertheless, rare Actinobacteria have been successfully isolated without pretreatments or complex culture media, including novel taxons. This is the case for isolate VN6-2, which presented the lowest 16S rRNA sequence similarity and forms a distinct phylogenetic branch, when compared to strains from the *Nocardiopsaceae* family. This novel strain was isolated with modified ISP-2, demonstrating that it is still worthwhile to use traditional cultivating methods for isolating novel Actinobacteria.

It is widely accepted that the discovery of new microorganisms are a good resource for the discovery of new bioactive compounds. In fact, a novel peptidic antibiotic derived from a novel genus has been recently discovered (Ling et al., 2015), which is a new cell wall inhibitor with promising activity against pathogenic bacteria. In our study, the phylogenetic analysis as well as the low 16S rRNA gene sequence similarity of isolate VN6-2 provides evidence that this isolate is a distinct genus that is related with the *Nocardiopsaceae* family. This is in agreement with the rational taxonomic boundaries proposed for high taxa of bacteria, using 16S rRNA gene sequences (Yarza et al., 2014). Depending on the culture media, we observed antimicrobial activity on selected Gram positive (*L. monocytogenes*) and Gram negative (*E. coli*) bacteria. Future efforts will be directed to investigate these activities as well as its exact taxonomic position.

One of the aspects of our work was to establish novel bacterial sources for antibiotic discovery. Ten of the twenty isolates tested showed antimicrobial activities. Under the cultivating conditions tested, two streptomycete isolates showed moderate inhibition (less than half of the bacterial line was inhibited in the antimicrobial screening test). Since the production of antimicrobial compounds can be influenced by different factors, including the nature of the cultivation media, pH and nutrient availability, appropriate cultivation methods should be addressed to further exploit these antibacterial activities. It is noteworthy to highlight the moderate to strong antibacterial activity (Table 2) shown of isolates VO29-3 (*Gordonia* sp.) belonging to the suborder *Corynebacterineae* (Figure 3), VP3-3 (*Isoptericola* sp.), VP2-3 (*Microbacterium* sp.), and VO30-3 (*Arthrobacter* sp.) belonging to the suborder *Micrococcineae* (Figure 3). To our knowledge, antibacterial activities from these genera have received scarce attention. Antarctic-derived *Arthrobacter* strains that showed activity against strains of the *Burkholderia cepacia* complex through the production of volatile organic compounds (Fondi et al., 2012; Orlandini et al., 2013; Papaleo et al., 2013). A *Microbacterium* sp. isolated from red algae, has been shown to have antimicrobial properties against both Gram negative and Gram positive bacteria (Kanagasabhapathy et al., 2008). In addition, antimicrobial activity of *Microbacterium* and *Gordonia* strains were reported (Graça et al., 2013). In this study, for the first time an antibacterial activity in an *Isoptericola* strain is reported. Recently, two *Isoptericola* strains isolated from subseafloor sediments that showed no antimicrobial activity were described (Ulanova and Goo, 2014). Further experiments will be carried out to deepen our knowledge on the antibacterial activities of these novel isolates.

The present study showed a rich biodiversity of culturable Actinobacteria from marine habitats of the Valparaíso coast in Central Chile. The 20 selected isolates were grouped into 18 phylotypes representing 16 families belonging to seven suborders. Interestingly, 10 of these isolates showed antibacterial activity. The biodiversity of the novel isolates from four suborders represent a valuable resource for the discovery of biologically active compounds and biotechnological applications.

### Conflict of interest statement

The authors declare that the research was conducted in the absence of any commercial or financial relationships that could be construed as a potential conflict of interest.

## Supplementary material

Figure S1 **Distribution of actinobacterial isolates in sediment samples from site 1 (Punta Ángeles Lighthouse) and site 2 (Torpederas Beach) of Valparaíso bay**. Site 1 comprises samples V1, V2, and V3, whereas site 2 comprises samples V4, V5, and V6.
