# Supplementary figures and images for: Culturable diversity and antimicrobial activity of Actinobacteria from marine sediments in Valparaíso bay, Chile
Source: Front Microbiol. 2015 Jul 28;6:737. doi: 10.3389/fmicb.2015.00737 (PMC4516979; doi:10.3389/fmicb.2015.00737)

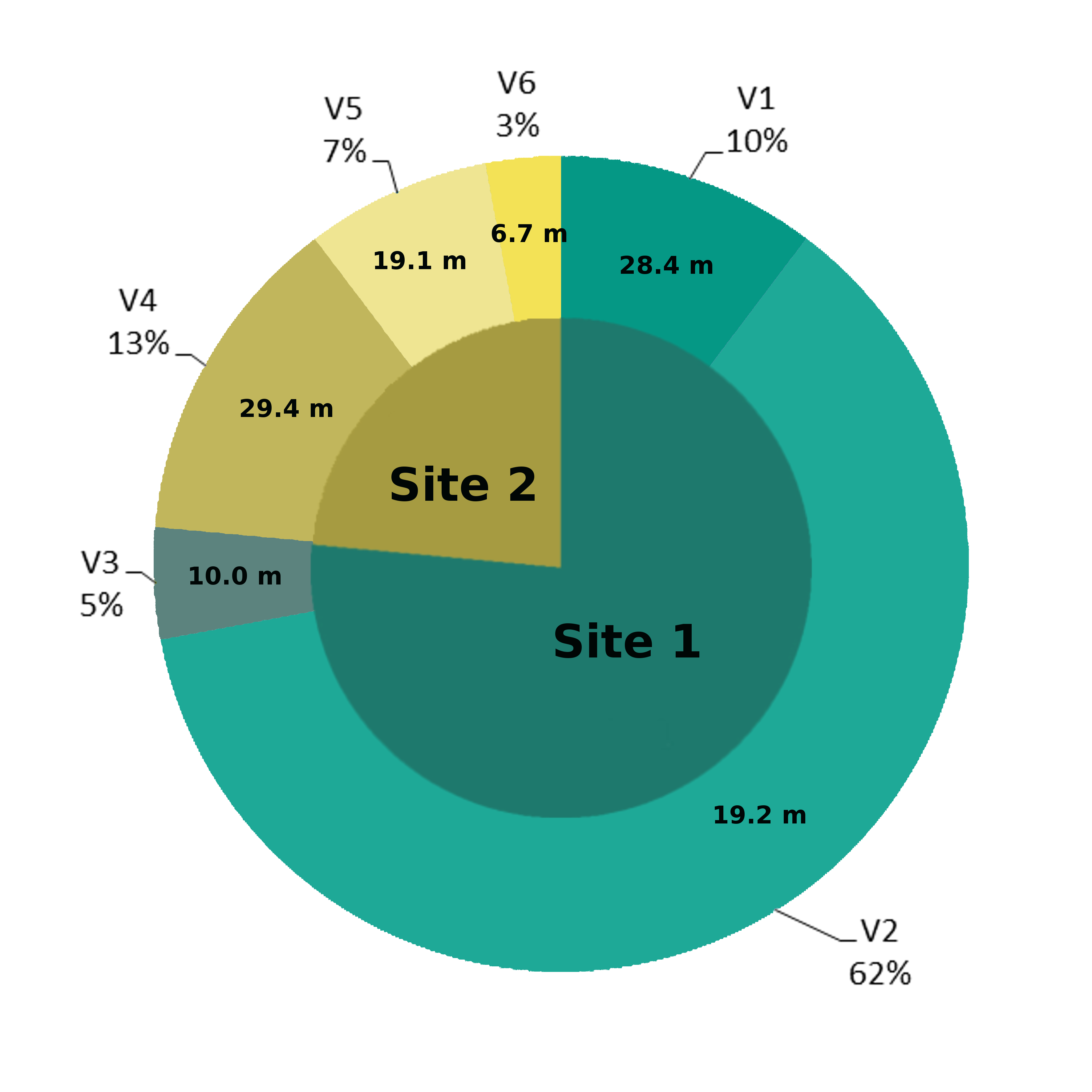

Supplement: Figure S1 — Distribution of actinobacterial isolates in sediment samples from site 1 (Punta Ángeles Lighthouse) and site 2 (Torpederas Beach) of Valparaíso bay. Site 1 comprises samples V1, V2, and V3, whereas site 2 comprises samples V4, V5, and V6. [file Image1.TIF]
